# Interesting Structures: Education and Outreach at the RCSB Protein Data Bank

**DOI:** 10.1371/journal.pbio.0060117

**Published:** 2008-05-06

**Authors:** Christine Zardecki

## Abstract

The Research Collaboratory for Structural Bioinformatics Protein Data Bank is part of an international consortium that works not only to make the data describing the 3-D shape of biological macromolecules available worldwide, but also to increase their usefulness in science, medicine, and education.

Knowing the shape of a protein can tell you its story—what it does and what molecules it binds with. Accordingly, understanding the structure of a biomacromolecule can help to create drugs designed to interact with a protein, either to block its access to other molecules or to prevent it from functioning. The Research Collaboratory for Structural Bioinformatics Protein Data Bank (RCSB PDB) is part of an international consortium that works to make the data describing the 3-D shape of biological macromolecules available worldwide. Additionally, the RCSB PDB creates resources and participates in outreach efforts to ensure that these data are used by scientists, students, and teachers, both inside and outside the structural biology community.

## The Protein Data Bank Archive

A new era of scientific research began when the 3-D shape of the oxygen-storing protein myoglobin was revealed [1,2]. Using x-ray crystallographic methods (and later nuclear magnetic resonance and cryo-electron microscopy), structural biologists have been elucidating the 3-D structures of proteins and nucleic acids ever since.

The types of experiments used to solve these structures produce a large amount of information, from the conditions and methods used to determine the molecule's structure to thousands of *x-y-z* coordinates describing the location of every atom viewed in the molecule. In the 1970s, structural biologists thought that they could benefit by sharing this information with one another, and the PDB archive, featuring seven structures, was born [3,4]. Since its inception, structural biologists from around the world have deposited and accessed the data contained in this archive, which is currently maintained by the Worldwide Protein Data Bank organization (wwPDB;http://www.wwpdb.org/) [5].

The online archive now contains more than 50,000 structures, which exhibit an amazing diversity of size, complexity, and function. Just a small and random sample of the biological macromolecules found in the PDB includes RNA, insulin, anthrax, influenza proteins, ribosomes, and prions. Rather than simply offering a data bank developed and used only by structural biologists, each wwPDB site offers resources and tools to access, view, and understand these data. The RCSB PDB maintains the master copy of the FTP archive for the wwPDB, and provides a comprehensive database and Web site at http://www.pdb.org/ [6]. These valuable resources are intended for a diverse group of users, including academic and industrial scientists, students and educators, science illustrators, textbook authors, media writers, and the general public. To address the very different needs of these users and ensure that RCSB PDB resources meet the specific needs of each group, our outreach and education initiatives must seek input from our diverse constituency. Only through interactions with all of these user groups will we be able to provide a resource that opens up the data contained in the PDB for applications in science, medicine, and education.

## Scientific Communities

While there isn't a “typical” user of the RCSB PDB resource, there are broad, consistent groups of people who are looking for different types of tools and resources. For example, our depositor community—the scientists who provide the data—use RCSB PDB tools and resources that aren't known to a significant portion of people who come to our Web site. Depositors are provided with their own help desk (deposit@deposit.pdb.org), Web site, tutorials, and tools. In developing our resources, it is very important to consider the types of information depositors can provide to the archive. Our services are customized by experimental type to facilitate the deposition process and to make sure that we are capturing the most data possible, as accurately as possible. To keep current, we collaborate with experts in various structural biology disciplines at meetings, in the development of data dictionaries, and on a variety of projects. As new methods in structure determination are being developed, we will meet and have workshops with the scientists who are generating these data, along with representatives of our data users to develop data content standards.

Another category, our “users,” includes the many people who use the RCSB PDB to access information, generate reports, and visualize structures. They could be searching for a structure that was recently published in a journal; looking for a group of structures that all bind to the same drug; or wanting to make an image containing the active site of a particular structure. Knowing that our users come to the site with varying degrees of expertise and knowledge, we try to provide a lot of information to guide any query up front; a help system is available from every page of the resource, along with printed and online Flash tutorials. An additional help desk (info@rcsb.org) answers queries from our non-depositors. Input from this help desk, in addition to other ways of gathering feedback, is used to develop tools that help the wide variety of people studying the structures of biological macromolecules and their relationships to sequence, function, and disease. A goal of the project is to develop resources that meet the needs of all users—from the structural genomist to the systems biologist and beyond.

For all of our users, it is extremely important to guarantee that the lines of communication are open both ways. While we try to communicate through weekly news, a quarterly newsletter, flyers, annual reports, and other resources, we realize that these publications may not be on every user's radar. A major focus of our outreach efforts has been to meet with users to learn how they are using RCSB PDB services and to identify any new enhancements or features that would better serve their methods and goals (see Table 1). We also actively participate in meetings to demonstrate RCSB PDB services and gather feedback, and publish in journals targeted to specific professional societies to inform users of new developments.

**Table 1:**
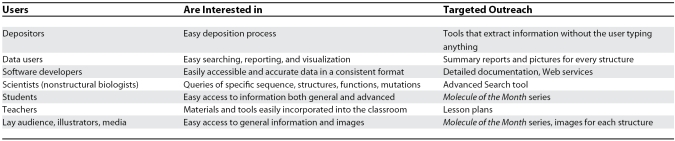
Selected Examples of RCSB PDB Users and Ongoing Outreach

## Students and Teachers

The helical structure of DNA has become so familiar in popular culture that it has been taken out of textbooks and placed onto jewelry, business logos, and was even featured in the opening ceremonies of the 2004 Olympics. Whether abstracted as two wavy lines or illustrated with detailed bases, the image of 3-D DNA is now widely viewed as representing “the building blocks of life.” The shapes of other structures, however, are not commonly known. Students may see a cloverleaf and be able to relate how messenger RNA binds to anticodons, but then see an L-shaped molecule and not recognize it as the same structure––transfer RNA.

As part of two universities, we promote our resources as a tool for education and research in courses and undergraduate research experiences locally. However, we also want our education efforts to reach younger students, their teachers, and the general public. Luckily, there are some features inherent in PDB structures that are able to attract this diverse audience:

*Proteins (and nucleic acids) are pretty*: Using the many amazing visualization programs available, we can see the beauty inherent in these structures. We exploit this fact when developing all materials, but specifically in our traveling *Art of Science* exhibit. By presenting large-scale images of proteins in the context of “art,” we have been able to informally educate students and teachers about the PDB resource.

**Figure 1:**
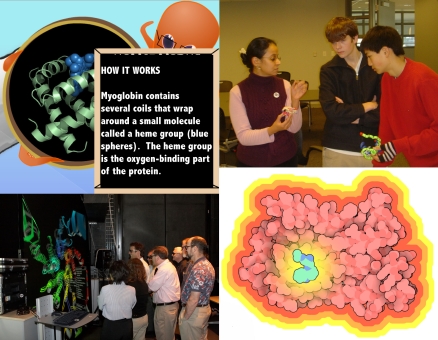
Examples of Education and Outreach Starting clockwise, from the upper left: A portion of an interactive kiosk highlighting proteins found in sea creatures that was part of an exhibit at the Birch Aquarium (La Jolla, California); the RCSB PDB's Shuchismita Dutta uses a computer-generated model to talk about protein structure with high school students at the New Jersey Science Olympiad; adrenergic receptors are the subject of the 100th *Molecule of the Month* installment; the RCSB PDB has developed a number of ways to explore structure, including this viewer for virtual reality environments.

Beautiful representations of structures are also a feature of the *Molecule of the Month* series (Figure 1). Written and illustrated by award-winning molecular biologist and illustrator David S. Goodsell (The Scripps Research Institute), each installment describes the structure and function of a molecule within the context of human health. *Molecule of the Month* is a starting point for several educational initiatives, including the Science Olympiad protein modeling event designed by the Center for BioMolecular Modeling (http://www.rpc.msoe.edu/cbm/scienceolympiad/) and run in New Jersey by the RCSB PDB. At this event, students build a model that highlights what makes the structure interesting using the *Molecule of the Month* entry, the PDB file, and visualization tools.

*Proteins are related to health and disease*: Though nutrition is the first thing that comes to mind when many people hear the word “protein,” we can build upon students' existing familiarity and interest in health and disease by selecting proteins related to these topics to exemplify structure–function relationships. In working with middle school and high school students, we have focused on the shape of virus structures in presentations and lessons. When building 3-D models of viruses, we discuss colds and what happens at the molecular level when we get sick. Viruses are particularly interesting, because their icosahedral shape easily lends itself to discussions about geometry and symmetry. At the collegiate level, we have explored and mapped structures in the context of their location in the body, the biological processes they participate in, and related diseases and disorders.

*The RCSB PDB resource is used by scientists worldwide*: When using the RCSB PDB, students are accessing the same Web site, database, and data used by researchers around the globe. Resources on the Web site, including the *Molecule of the Month*, visualization tools, and links to other authoritative scientific sites, provide interactive opportunities to increase scientific and computing literacy and understanding of how researchers view structural data. An early introduction to these tools and information can get students to start thinking like the scientists who use them.

We are always trying to think of new ways to interest teachers and students. After speaking with teachers at workshops, conferences, and other events, we recognized that hands-on activities and lesson plans would be useful tools to start teaching about structure. The addition of these features has been received enthusiastically, and has led educators to suggest additional materials. We are able to spark student interest with temporary tattoos and pins of collagen and other structures that are given away at school events and conferences, and then go a step further by teaching key points about molecular structure, having students use the tools and resources available at the Web site and build hands-on 3-D models of what they can see online.

## The Shape of Things to Come

With so many varied types of outreach, we internally emphasize tracking the reactions of all of our audiences. We've seen a growing sense of familiarity with our resources at the various professional society meetings, and are excited that many students are able to list proteins that they'd like to learn more about.

Based on feedback from our users, selected future plans include packaging our lesson plans for a larger distribution and creating materials to introduce the nonstructural biologist to PDB data. If you have comments about our resources, things that you would like to see at the RCSB PDB Web site, or questions about the 3-D structures of proteins and nucleic acids, please let us know at info@rcsb.org.

## Abbreviations

RCSB PDB: Research Collaboratory for Structural Bioinformatics Protein Data Bank
wwPDB: Worldwide Protein Data Bank
